# Dense Sm and Mn Co-Doped BaTiO_3_ Ceramics with High Permittivity

**DOI:** 10.3390/ma12040678

**Published:** 2019-02-25

**Authors:** Qiaoli Liu, Junwei Liu, Dayong Lu, Tingqu Li, Weitao Zheng

**Affiliations:** 1 Key Laboratory for Special Functional Materials in Jilin Provincial Universities, Jilin Institute of Chemical Technology, Jilin 132022, China; liuqiaoli234@163.com (Q.L.); jwju@foxmail.com (J.L.); ltq2000@163.com (T.L.); 2 School of Materials Science and Engineering, Key Laboratory of Mobile Materials, MOE, and State Key Laboratory of Superhard Materials, Jilin University, Changchun 130012, China

**Keywords:** BaTiO_3_, solid state reaction, valence state, electron paramagnetic resonance, high permittivity

## Abstract

The structure, valence state, and dielectric properties of (Ba_1−*x*_Sm*_x_*)(Ti_0.99_Mn_0.01_)O_3_ (BSTM) (*x* = 0.02‒0.07) ceramics prepared via a high temperature (1400 °C/12 h) solid state reaction were investigated. A homogeneous and dense microstructure was observed in all samples. With increasing Sm content, the crystal structure changed from tetragonal (*x* ≤ 0.06) to cubic (*x* = 0.07) and unit cell volume (*V*_0_) decreased continuously, which was mainly due to the substitution of Ba^2+^ ions by smaller Sm^3+^ ions in the perovskite lattice. Electron paramagnetic resonance investigation revealed that Mn ions were reduced from high valence to low valence under the role of Sm^3+^ donor, and only Mn^2+^ ions were observed at *x* = 0.07. The Curie temperature (*T*_c_) moved to lower values, from 105.5 down to 20.4 °C, and the *x* = 0.07 sample satisfied Y5V specification with high permittivity (*ε*′_RT_ > 13,000) and low loss (tan δ < 0.03).

## 1. Introduction

Transition metal Mn-doped BaTiO_3_ ceramics are widely applied in ceramic capacitors [1,2] and positive temperature coefficient of resistance (PTCR) devices [3,4]. Because of its small ionic radius, Mn occupies the octahedral Ti site as an acceptor and occurs in variable states of Mn^2+^, Mn^3+^, and Mn^4+^, which are commonly accepted in the literature [1,2,3,4,5,6]. Mn is found to have the significant effect on the reduction of dielectric loss (tan *δ*) [2,5,7] and the increase of resistivity [1,3,4]. Jayanthi and Kutty [2] reported that the room temperature tan *δ* was 0.009 for 0.75 at % Mn-doped BaTiO_3_ ceramics, and only 0.002 for 1 at % Mn. Lu et al. [8] found that tan *δ* of Ba(Ti_0.99_Mn_0.01_)O_3_ was less than 0.02 in the temperature range from −70 to 200 °C. The lower value of tan *δ* for BaTiO_3_ ceramics containing Mn implies that the Mn acceptor has a high ability to conduct electron trapping. This was confirmed by Wang et al. [9]. In their study, the authors proposed that Mn^3+^ and Mn^4+^ on the Ti sites could effectively capture the conductance electrons, leading to the drop of carrier concentration in materials. 

At the same time, with the increase of Mn content a decrease in dielectric permittivity is also observed [2,5,7], which can be ascribed to the formation of a paraelectric hexagonal phase. According to the earlier studies carried out in the air or in an oxidizing atmosphere, Mn is the most effective dopant for stabilizing hexagonal BaTiO_3_ (h-BT) at room temperature [10], and the existence of h-BT is attributed to the Jahn–Teller distortion (i.e., Jahn–Teller effect) caused by ${\mathrm{Mn}}_{\mathrm{Ti}}^{3+}$ [1]. Such a structure is not desirable in the practical application of ceramic capacitors, since h-BT has a lower permittivity [11]. Our study has found that hexagonal Ba(Ti_0.97_Mn_0.03_)O_3_ exhibits a low permittivity of about 100 at room temperature. 

It is known that the addition of rare earth ions can modify the dielectric properties of BaTiO_3_ [12,13,14,15,16]. In Mn-doped BaTiO_3_ ceramics the partial replacement of Ba^2+^ by rare earth ions is reported to improve the dielectric properties and inhibit the formation of the undesired hexagonal phase. Our previous studies on Nd and Mn co-doped BaTiO_3_ ceramics have revealed that the addition of Nd^3+^ can significantly inhibit the hexagonal phase and lead to an obvious improvement of dielectric properties in Mn-doped BaTiO_3_ ceramics [17]. Gong et al. [18] reported that in Ba_1−*x*_La*_x_*Ti_0.96_Mn_0.04_O_3_ ceramics, La^3+^ ions could suppress the formation of hexagonal phase effectively. A similar phenomenon was also observed in Ba_1−*x*_Pr*_x_*Ti_0.99_Mn_0.01_O_3_ ceramics [8]. 

In this work, Sm and Mn were co-doped into BaTiO_3_ to improve the dielectric properties of BaTiO_3_ ceramics, that is, to obtain high dielectric permittivity and low dielectric loss at the same time. Besides the structure and dielectric properties of (Ba_1−*x*_Sm*_x_*)(Ti_0.99_Mn_0.01_)O_3_ (BSTM), the valence states of Mn ions, which are closely related to the crystal structure, are also discussed.

## 2. Experimental Section

### 2.1. Sample Preparation

Analytical-grade BaCO_3_ (99.4%), TiO_2_ (99.5%), Sm_2_O_3_ (99.9%), and MnO_2_ (99%) powders were used to prepare (Ba_1−*x*_Sm*_x_*)(Ti_0.99_Mn_0.01_)O_3_ (BSTM) ceramics by a solid-state reaction method, where *x* = 0.02, 0.04, 0.06, and 0.07. The stoichiometric amounts of raw powders were grinded in an agate mortar for about 1 h, and then calcined at 1100 °C for 5 h. The calcined powders were pressed into disks of 12 mm diameter at 200 MPa pressure after mixing with polyvinyl alcohol solution. The final sintering was performed at 1400 °C for 12 h under air atmosphere in an electric furnace. Heating was conducted at a rate of 100 °C/h, and cooling was conducted at a rate of −200 °C/h until 1000 °C was reached, and was then followed by natural cooling to room temperature. As a comparison, (Ba_1−*x*_Sm*_x_*)Ti_1−*x*/4_O_3_ (*x* = 0.07) ceramic (abbreviated BS7T) was also prepared under the same conditions for the analysis of Raman bands at the high-wavenumber region. BSTM ceramics with *x* = 0.02, 0.04, 0.06, and 0.07 were abbreviated as BS2TM, BS4TM, BS6TM and BS7TM, respectively. The ceramic density was estimated by the Archimedes method. 

### 2.2. Characterization 

The surfaces of samples were mechanically polished and then thermally etched at 1400 °C for 15 min in air. Microstructures were observed using scanning electron microscopy (SEM) (EVOMA 10, Zeiss, Oberkochen, Germany) with secondary electron (SE) image and backscattered electron (BSE) images at an accelerating voltage of 15 keV. The average grain size (*GS*) was estimated using Fullman’s method [19], as described in our previous report [20].

Powder X-ray diffraction (XRD) using an X-ray diffractometer (DX-2700, Dandong Haoyuan, Dandong, China) with Cu Kα radiation was carried out at room temperature in order to characterize the crystal structure of the sintered ceramics. The data were collected between 20° ≤ 2θ ≤ 85°, with a step increment of 0.02° and a count time of 3 s. The crystal structure was determined by MS Modeling 4.0 software (Accelry Inc., San Diego, CA, USA) and Cu Kα_1_ radiation (λ = 1.540562 Å). 

Raman spectra were measured using a Raman spectrometer (XploRA, Horiba Jobin Yvon, Longjumeau, France) with excitations of 532 and 638 nm. A heating/cooling stage (THMS600, Linkam, Surrey, UK), which provided precise temperature control, was used for the temperature-dependent Raman scattering measurements. 

Electron paramagnetic resonance (EPR) spectra were recorded from 30 to 150 °C using an X-band (~9.8 GHz) EPR spectrometer (A300-10/12, Bruker, Rheinstetten, Germany) equipped with a temperature controller capable of stabilizing the temperature within less than 0.1 °C. The *g*-factor of the EPR signal was determined by the fundamental equation as shown below:

hν = *g*βH, (1)

where h is the Planck constant, ν is the microwave frequency, β is the Bohr magnetron, H is the magnetic field strength. In the experiment the microwave frequency and center field were ~9.4 GHz and 3500 G, respectively. 

Prior to dielectric measurements, the sintered samples were polished to 0.8 mm in thickness, and gold was sputtered and then silver was pasted on both sides in order to form electrodes. The dielectric properties as functions of temperature (−75–200 °C) and frequency (1–10^6^ Hz) were measured using a broadband dielectric spectrometer (Concept 41, Novocontrol, Montabaur, Germany). 

## 3. Results and Discussion

### 3.1. Structure

#### 3.1.1. SEM Observations

**Figure 1:**
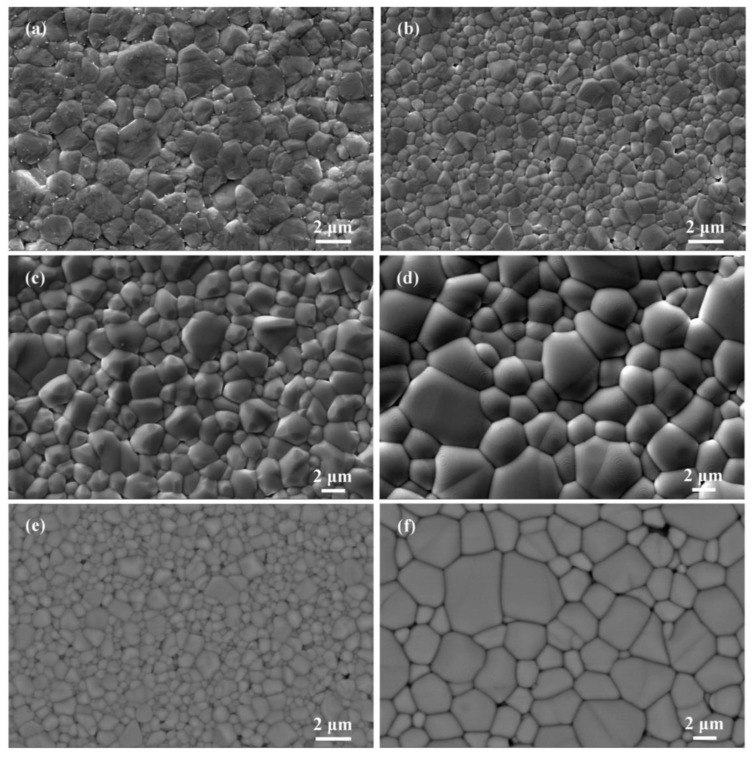
SEM images of (Ba_1−*x*_Sm*_x_*)(Ti_0.99_Mn_0.01_)O_3_ (BSTM) ceramics with (**a**) *x* = 0.02, (**b**) 0.04, (**c**) 0.06, and (**d**) 0.07, and BSE images of BSTM ceramics with (**e**) *x* = 0.04 and (**f**) 0.07.

The relative density (*ρ*_r_) of BSTM ceramics was about 93% with no clear dependence on dopant content, which was calculated using the following relation:

(2) $$\rho_{r}=\frac{\rho_{b}}{\rho_{t}}\times 100$$

where *ρ*_b_ is the ceramic bulk density, and *ρ*_t_ is the theoretical density. The values of *ρ*_b_, *ρ*_t_, and *ρ*_r_ are listed in Table 1. SE and BSE images of BSTM ceramics are shown in Figure 1. The homogeneous and dense microstructures are observed (Figure 1a–d), and the data of average grain size (*GS*) of specimens are tabulated in Table 1. The sample with *x* = 0.04 exhibited a fine-grained microstructure with *GS* = 0.8 μm. BSE images are helpful to obtain information about the chemical composition of a sample and to quickly distinguish the potential secondary phases. As shown in Figure 1e,f, no evident compositional inhomogeneity was observed, implying the doping was homogeneous; that is to say, Sm and Mn dopants were successfully incorporated into BaTiO_3_ without detectable secondary phases. 

#### 3.1.2. X-ray Diffraction Analysis

**Figure 2:**
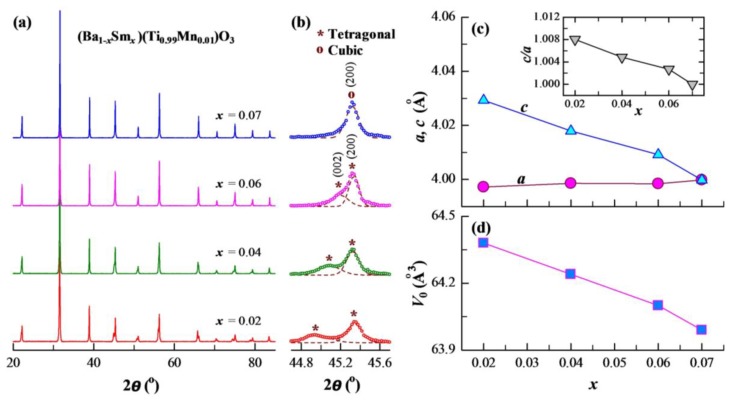
(**a**) Powder XRD patterns of BSTM ceramics. (**b**) Enlarged peaks in the vicinity of 45°; the dashed lines denote Gaussian fits of the peaks. (**c**) Lattice parameters (*a*, *c*) and (**d**) unit cell volume (*V*_0_) as a function of *x*. The inset depicts the tetragonality (*c*/*a*) as a function of *x*.

The powder XRD patterns and enlarged peaks in the vicinity of 45° of BSTM are shown in Figure 2a,b. All samples exhibited sharp and well-defined peaks, indicating the good crystallinity of BSTM ceramics. Comparing with the standard XRD patterns of tetragonal (JCPDS No. 05-0626) and cubic BaTiO_3_ (JCPDS No. 31-0174), all the diffraction peaks could be perfectly indexed to the tetragonal perovskite structure when *x* ≤ 0.06, as marked by the characteristic (002)/(200) peaks at ~45° (Figure 2b), and to the cubic structure when *x* = 0.07, as marked by a symmetric (200) peak (Figure 2b). In addition, there were no diffraction peaks of impurities, such as Sm or Mn oxides, suggesting that Sm and Mn ions completely entered into the BaTiO_3_ host lattice, which was consistent with the observations of BSE images. Lu et al. [8] confirmed that tetragonal and hexagonal phases coexisted in Ba(Ti_0.99_Mn_0.01_)O_3_, and the existence of the hexagonal phase resulted from the Jahn–Teller distortion caused by ${\mathrm{Mn}}_{\mathrm{Ti}}^{3+}$. However, our XRD results indicated that no diffraction peaks of the hexagonal phase could be detected in BSTM ceramics. In addition, no rod-like or plate-like hexagonal grains were observed in all samples, as shown in Figure 1. It can be concluded that Sm^3+^ ions can effectively inhibit the formation of the hexagonal phase, which was very similar to that obtained for (Ba_1−*x*_Pr*_x_*)(Ti_0.99_Mn_0.01_)O_3_ [8] and (Ba_1−*x*_Nd*_x_*)(Ti_0.97_Mn_0.03_)O_3_ [17]. A possible explanation for the absence of hexagonal phase is that for keeping charge neutrality, Mn^3+^ ions stabilizing the hexagonal phase can be reduced to Mn^2+^ ions under the role of Sm^3+^ donor, and $2{\mathrm{Sm}}_{\mathrm{Ba}}^{∙}-{\mathrm{Mn}}_{\mathrm{Ti}}^{\prime \prime }$ defect complexes are formed. For rare earth and Mn co-doped systems, the formation of donor–acceptor complexes, such as $2{\mathrm{Sm}}_{\mathrm{Ba}}^{∙}-{\mathrm{Mn}}_{\mathrm{Ti}}^{\prime \prime }$, will prevent Mn^2+^ from being oxidized, even in air atmosphere [17,21,22,23]. The valence state of Mn ions will be discriminated further using the EPR technique.

The lattice parameters (*a*, *c*) and unit cell volume (*V*_0_) were calculated by MS Modeling software, as plotted in Figure 2c,d, and the inset depicts the tetragonality (*c*/*a*) as a function of *x*. It can be seen that the lattice parameter *a* expanded slightly with increasing *x*, whereas the lattice parameter *c* decreased significantly. When *x* = 0.07, *a* was equal to *c*, and a cubic-phase ceramic was formed. The inset shows that *c*/*a* decreases, which is consistent with Figure 2b. The separate (002) and (200) peaks shifted toward each other, also indicating a decrease in tetragonality. The *V*_0_ of BSTM ceramics was less than that of tetragonal BaTiO_3_ (64.41 Å^3^) and decreased continuously with increasing *x*, indicating that Sm^3+^ ions mainly substituted for Ba sites instead of Ti sites. The replacement of Ba^2+^ (1.61 Å) [24] ions by smaller Sm^3+^ (1.24 Å) [24] ions caused the contraction of *V*_0_. It was consistent with the previous report that Sm ions preferentially occupy Ba sites at Ba/Ti < 1 [25]. Tsur et al. [26] have also reported that Sm incorporates mainly into Ba sites.

#### 3.1.3. Raman Scattering Investigations

**Figure 3:**
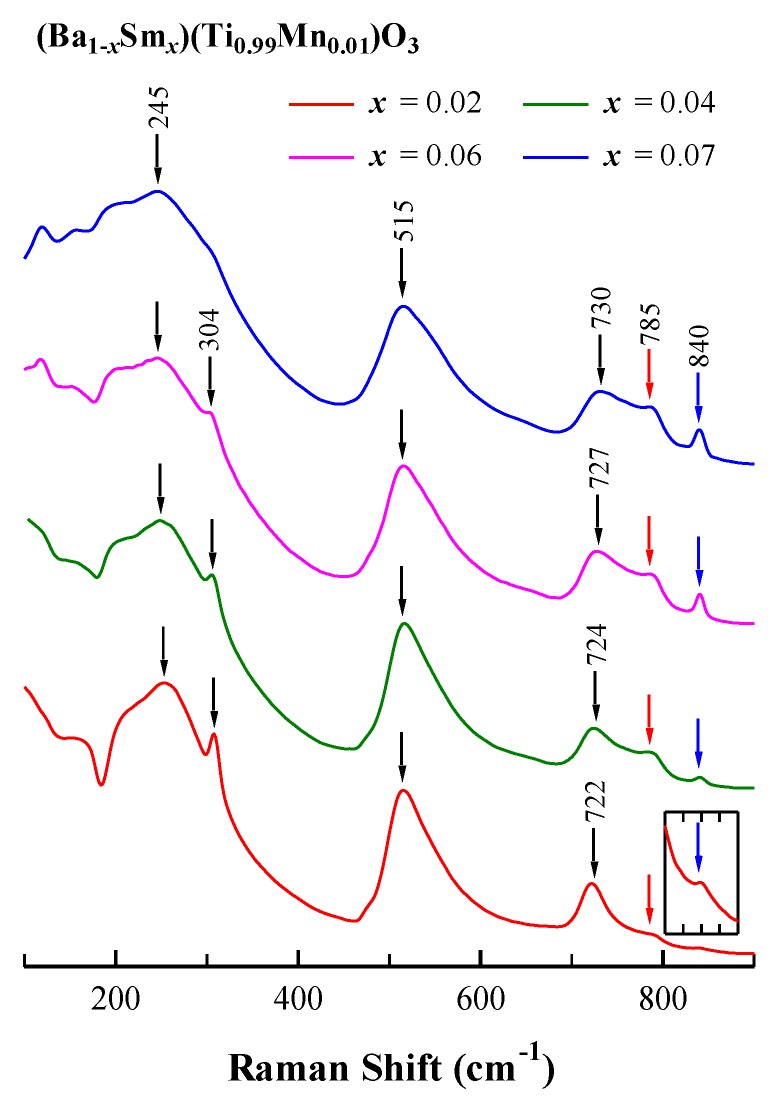
Raman spectra of BSTM ceramics excited by a 532 nm laser.

Together with the XRD studies, we employed Raman spectroscopy measurements to investigate the structure and phase transformation. Raman spectra of BSTM ceramics, excited by a 532 nm laser in the range of 100–900 cm^−1^ at room temperature, are shown in Figure 3. When *x* ≤ 0.06, six Raman bands at 245, 304, 515, 722, 785, and 840 cm^−1^ were observed, and the 245, 304, 515, and 722 cm^−1^ bands corresponded to tetragonal BaTiO_3_ [27,28]. The Raman band at 305 cm^−1^, which was assigned to B_1_+E(LO+TO) [28], was characteristic of the tetragonal phase [29]. This band gradually weakened, indicating a decrease in tetragonality, and disappeared at *x* = 0.07, suggesting a phase transition from tetragonal to cubic, which was in good accordance with the XRD results.

A noticeable shift in position for the band at 722–730 cm^−1^ was observed. We propose that doping with Sm^3+^ ions will induce the valence change of Mn ions from high valence to low valence for keeping charge neutrality. Since Mn^2+^ (0.67 Å) has a larger ionic radius than that of Mn^3+^ (0.58 Å) and Mn^4+^ (0.53 Å), the Mn–O bonds have higher covalency, and 722 cm^−1^ bands shift toward a higher frequency. A similar behavior was also found in Nd/Mn co-doped BaTiO_3_ [17]. 

**Figure 4:**
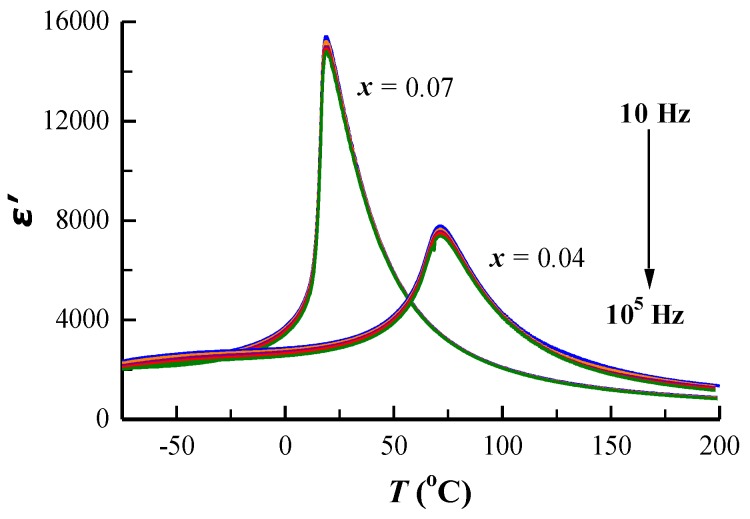
Temperature dependence of dielectric permittivity for samples with *x* = 0.04 and 0.07 at various frequencies.

**Figure 5:**
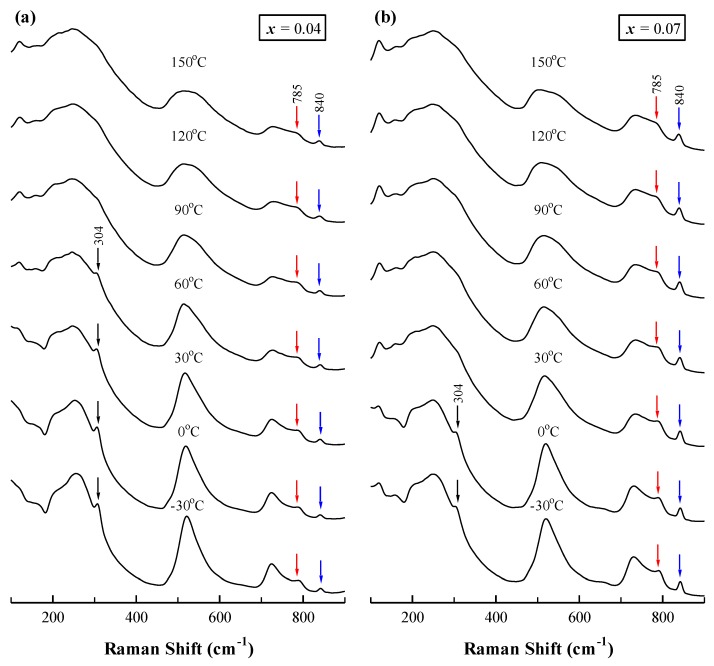
Temperature-dependent Raman spectra of samples with (**a**) *x* = 0.04 and (**b**) *x* = 0.07 excited by a 532 nm laser.

Two additional 785 and 840 cm^−1^ bands, which were not observed in BaTiO_3_ [27,28,29], appeared in all samples. The 785 cm^−1^ band was also observed in Ba(Ti_1−_*_x_*Zr*_x_*)O_3_ [30,31,32,33] and La_0.4_Ba_0.6_Ti_0.6_RE_0.4_O_3_ (RE = Y, Yb) [34] ceramics. Farhi et al. [30] claimed that this band was related to the occurrence of relaxor properties, but Pokorny et al. [33] and Feteira et al. [34] assigned it to the A_1g_ octahedral breathing mode. In order to investigate the origin of the 785 cm^−1^ band, the dielectric permittivity (*ε*′) of representative samples with *x* = 0.04 and 0.07 as a function of temperature at various frequencies (10–1 × 10^5^ Hz) is shown in Figure 4. The Curie temperature (*T*_c_) of BS4TM and BS7TM remained unchanged as the frequency increased. The result shows that there was no frequency dispersion in BSTM ceramics, which indicated that BSTM ceramics were not relaxor ferroelectrics, but normal ferroelectrics. Pokorny et al. [33] reported that the A_1g_ octahedral breathing mode was inactive in BaTiO_3_ since this mode is symmetrical. However, for BaTiO_3_-based ceramics with two or more B-site species, the A_1g_ band became Raman-active. The presence of different ions in the center of the BO_6_ octahedra created asymmetry in the breathing-like mode, and the width and intensity of this band was largely temperature-independent in the vicinity of the cubic-to-tetragonal phase transition [33]. Figure 5 shows the temperature-dependent Raman spectra from −30 to 150 °C of representative samples with *x* = 0.04 and 0.07 at the wavelength of 532 nm. It showed that the band at 785 cm^−1^ was almost independent of temperature, as reported in the literature. Therefore, we preferred to assign the 785 cm^−1^ band to A_1g_ octahedral breathing mode. In addition, the 304 cm^−1^ band representing the tetragonal structure disappeared above 60 and 0 °C for *x* = 0.04 and 0.07 samples, respectively, indicating that the tetragonal-to-cubic transition temperature (i.e., *T*_c_) decreased with increasing Sm content.

Figure 3 shows that the intensity of the 840 cm^−1^ band increased gradually with Sm content. We attributed this band to the Raman charge effect, which arose from an internal deformation of the BO_6_ octahedron induced by the occupation of trivalent ions (La^3+^, Nd^3+^, Sm^3+^, etc.) at equivalent Ba sites in the BaTiO_3_ lattice [35]. The 840 cm^−1^ band was first reported in La-doped BaTiO_3_ by Kchikech and Maglione [36], and its intensity was proportional to the La content. Mazon et al. [37] also observed the appearance of a new mode at ~850 cm^−1^ in Nd-doped Ba_0.77_Ca_0.23_TiO_3_ ceramics, and the intensity of this mode increased linearly with Nd content. Figure 5 shows that the 840 cm^−1^ band did not change as a function of temperature, and persisted up to 150 °C. It indicated that the tetragonal–cubic phase transition did not affect the Raman charge effect, as reported in the literature [38]. 

**Figure 6:**
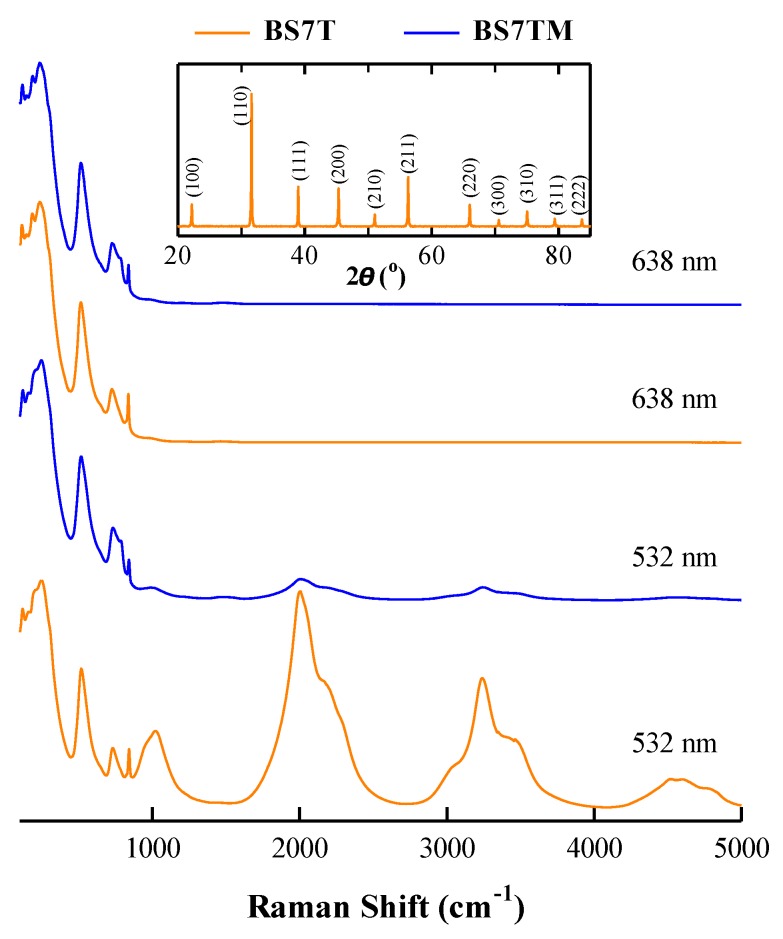
Room temperature Raman spectra of BS7T and BS7TM ceramics excited by 532 and 638 nm lasers. The inset shows the XRD pattern of BS7T.

Figure 6 exhibits room temperature Raman spectra of the representative samples BS7T and BS7TM excited by 532 and 638 nm lasers in the wide wavenumber range 100–5000 cm^−1^. BS7T ceramic with a cubic structure (Figure 6 inset) was prepared under the same conditions (1400 °C/12 h) as BSTM ceramics. Abnormal Raman spectra excited by the 532 nm laser were observed at a Raman shift > 1000 cm^−1^ in BS7T and BS7TM. However, these bands were not observed at the 638 nm excitation wavelength. Lu et al. [39] observed similar abnormal Raman spectra in Er-doped BaTiO_3_ ceramics, and using different excitation wavelengths, they confirmed that the abnormal Raman spectra originated from a fluorescent effect of Er^3+^. Gajović et al. [40] succeeded in observing the luminescence bands in Y_2_O_3_ powders by Raman spectrometer with visible excitations of 514.5 and 488.1 nm. Here, the abnormal Raman spectra in BS7T and BS7TM were very similar to the fluorescent spectra of Sm-doped phosphors [41]. Therefore, the bands at > 1000 cm^−1^ were related to a fluorescent effect of Sm^3+^. And the formation of $2{\mathrm{Sm}}_{\mathrm{Ba}}^{∙}-{\mathrm{Mn}}_{\mathrm{Ti}}^{\prime \prime }$ donor-acceptor defect complexes in BSTM led to a significant decrease in fluorescent intensity of Sm^3+^ at the 532 nm excitation wavelength.

### 3.2. Valence State of Mn Ions

**Figure 7:**
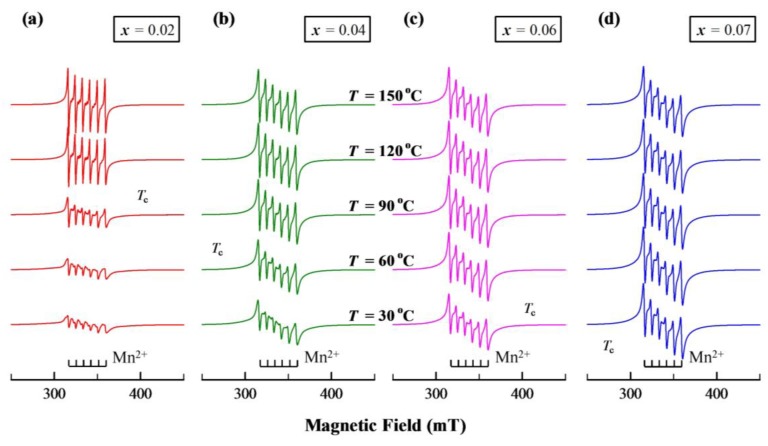
Electron paramagnetic resonance (EPR) spectra of BSTM ceramics with (**a**) *x* = 0.02, (**b**) *x* = 0.04, (**c**) *x* = 0.06, and (**d**) *x* = 0.07 at different temperatures.

The crystal structure of BaTiO_3_ doped with Mn is closely related to the valence state of Mn [1,42]. Different valences of Mn ions in BSTM were assessed by EPR. The variable temperature X-band EPR spectra of BSTM are shown in Figure 7. 

As seen from the EPR spectra at 30 °C, a clear sextet signal with *g* ≈ 2.0 was observed in all samples. The sextet signal was also observed in BaTiO_3_ containing Mn^2+^ [43,44]. Mn^2+^ (3*d*^5^) as a Kramer ion is EPR-active, and showed the sextet hyperfine structure of ^55^Mn (I = 5/2) [44,45]. In Ba(Ti_0.99_Mn_0.01_)O_3_ ceramics with mixed tetragonal and hexagonal phases, the coexistence of Mn^3+^ and Mn^4+^ was determined by EPR technique [8], and the hexagonal phase resulted from the Jahn–Teller distortion caused by ${\mathrm{Mn}}_{\mathrm{Ti}}^{3+}$ [1]. Because no diffraction peaks of the hexagonal phase were detected in BSTM ceramics (Figure 2), there were no Mn^3+^ ions in all samples. Here, the sextet signal was noticed in all samples, indicating that the Sm^3+^ donor induced the conversion of high valence state Mn to divalent Mn. Thus, Mn ions existed as two valence states, Mn^2+^ and Mn^4+^, in BSTM with a tetragonal structure (*x* ≤ 0.06). This result was in good agreement with previous experimental results. Wang et al. [46] confirmed that Mn^2+^ and Mn^4+^ coexisted in MnCO_3_-modified (Ba_0.85_Ca_0.15_)(Zr_0.1_Ti_0.9_)O_3_ ceramics with tetragonal phase using XPS.

It was observed that the intensity of the sextet signal from Mn^2+^ increased with increasing Sm content. Thus, it could be inferred that more Mn^4+^ ions converted to Mn^2+^. Meanwhile, the crystal structure changed from tetragonal to cubic. We concluded that Mn^2+^ ions contributed to tetragonal-to-cubic transformation. Kirianov et al. [42] studied the effect of Mn ions on the BaTiO_3_ crystal structure under different oxygen pressures. They found that Mn^2+^ ions promoted the transfer of BaTi_1−*x*_Mn*_x_*O_3_ from the tetragonal phase to the cubic phase in a reducing atmosphere. The hyperfine sextet of Mn^2+^ was independent of temperature at *x* = 0.07, as shown in Figure 7d, which implied that all of the Mn ions existed as Mn^2+^ in BS7TM with cubic symmetry. In addition, the tetragonal-to-cubic transition temperature *T*_c_ can be identified by the temperature-dependent EPR spectra, which were consistent with the results in Figure 5.

### 3.3. Dielectric Properties

**Figure 8:**
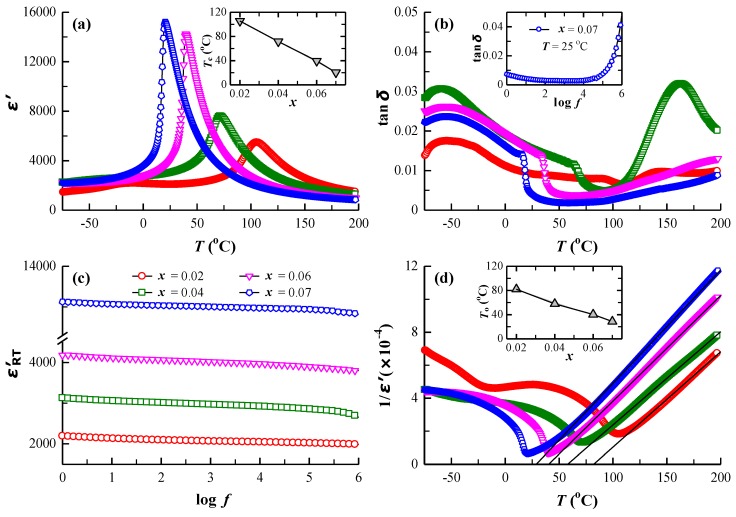
(**a**) Dielectric permittivity (ε′) and (**b**) dielectric loss (tan δ) as functions of temperature for BSTM ceramics. The insets depict *T*_c_ versus *x* and frequency dependence of room temperature tan *δ* for the *x* = 0.07 sample; (**c**) variation of room temperature permittivity (*ε*′_RT_) as a function of frequency for BSTM ceramics; (**d**) curves of 1/ε′ versus temperature at 1 kHz (symbols, experimental data; solid line, fitting to the Curie–Weiss law). The inset depicts *T*_0_ versus *x*.

All samples were electrical insulators with a resistivity of *ρ* > 10^10^ Ω cm at room temperature. The dielectric permittivity (*ε*′) and dielectric loss (tan *δ*) as functions of temperature for BSTM measured at 1 kHz are shown is Figure 8a,b. The dielectric permittivity exhibited a peak at Curie temperature (*T*_c_), which was related to a ferroelectric (tetragonal)–paraelectric (cubic) phase transition. With increasing Sm content, the maximum permittivity (*ε*′_m_) increased from 5480 to 15,220 and the room temperature permittivity (*ε*′_RT_) increased from 2080 to 13,810. The Curie temperature *T*_c_ decreased to room temperature, and the sample with *x* = 0.07 satisfied the EIA Y5V specification (i.e., (*ε*’ − *ε*’_RT_)/*ε*’_RT_ within +22 to −82% in the temperature range of −30 to 85 °C) with tan δ < 0.03. Cai et al. [47] reported that the occupation of Sm^3+^ ions on Ba sites in BaTiO_3_ led to the fall of *T*_c_. Park et al. [14] prepared Sm-doped BaTiO_3_ ceramics using the liquid-mix method and solid-state reaction, and found that *T*_c_ moved to lower temperatures with the increase of Sm content. The Figure 8b inset shows that room temperature tan *δ* was less than 0.05 over a range from 1 to 10^6^ Hz for *x* = 0.07. In addition, the Curie temperature determined by dielectric measurements was in good agreement with the temperature range of the phase transition presented in variable temperature Raman (Figure 5) and EPR spectra (Figure 7). Figure 8c shows the frequency dependence of *ε*′_RT_ in the wide frequency range of 1–1 × 10^6^ Hz. It was discovered that *ε*′_RT_ was essentially independent of frequency, similar to our previous work of BaTiO_3_ ceramics co-doped with Nd and Mn [17]. It is known that ionic displacement polarization can maintain the permittivity until a high frequency; however, the space charge polarization occurs only at low frequencies. Therefore, the ionic displacement polarization plays a dominate role in BSTM ceramics, and the effect of space charge can be neglected, which may be ascribed to the formation of $2{\mathrm{Sm}}_{\mathrm{Ba}}^{∙}-{\mathrm{Mn}}_{\mathrm{Ti}}^{\prime \prime }$ defect complexes.

Furthermore, the dielectric properties of a normal ferroelectric material meet the Curie–Weiss law described by the equation as below:

1/*ε*′ = (*T* − *T*_0_)/*C* (*T* > *T*_c_), (3)

where *T*_0_ and *C* denote the Curie–Weiss temperature and the Curie constant, respectively. Figure 8d presents the inverse dielectric permittivity as a function of temperature at 1 kHz. With increasing Sm content, *T*_0_ decreased from 82.0 to 28.7 °C in a manner similar to *T*_c_, as shown in the Figure 8d inset. The Curie constant *C* increased at first, and then decreased. BS4TM exhibited the highest value of C. It is known that the value of *C* is related to the grain size and porosity of samples [22]. All samples showed dense microstructures, and BS4TM exhibited the smallest grain size, so the value of C measured in BS4TM was higher than that of other samples. Table 2 summarizes the dielectric parameters for BSTM ceramics at a frequency of 1 kHz, including maximum permittivity *ε*′_m_, room temperature permittivity *ε*′_RT_, Curie temperature *T*_c_, Curie–Weiss temperature *T*_0_, and the Curie constant *C*.

## 4. Conclusions

Sm and Mn co-doped BaTiO_3_ ceramics with different Sm_2_O_3_ contents, ranging from 2 to 7 at % Sm, were synthesized by a conventional solid-state reaction sintering at 1400 °C in air for 12 h. The content of 1 at % Mn was constant in all samples. A homogeneous and dense microstructure was observed in all samples with an average grain size ranging from 0.8 to 3.5 μm. XRD and RS analysis revealed that the samples had a single-phase tetragonal structure when *x* ≤ 0.06 and a cubic structure at *x* = 0.07. With increasing Sm content, the unit cell volume was found to decrease continuously, confirming the substitution of Ba^2+^ ions by smaller Sm^3+^ ions in the perovskite lattice. EPR investigation revealed that Mn ions exhibited the mixed valence states of Mn^2+^ and Mn^4+^ in a tetragonal phase (*x* ≤ 0.06), and all of the Mn ions existed as Mn^2+^ in a cubic phase (*x* = 0.07). The dielectric properties were investigated as functions of frequency (1–1 × 10^6^ Hz) and temperature (−75 to 200 °C). The Curie peak shifted towards a lower temperature, and the *x* = 0.07 sample satisfied EIA Y5V specification with *ε*′_RT_ = 13,810 and tan δ < 0.03. BSTM ceramics as normal ferroelectrics exhibited a near frequency-independent stability over a frequency range of 1–1 × 10^6^ Hz, implying that ionic displacement polarization plays a dominate role in BSTM ceramics.

## Figures and Tables

**Table 1:** Values of *ρ*_b_, *ρ*_t_, *ρ*_r_ and average grain size (*GS*) for (Ba_1−*x*_Sm*_x_*)(Ti_0.99_Mn_0.01_)O_3_ ceramics.

Composition *x*	*ρ*_b_ (g/cm^3^)	*ρ*_t_ (g/cm^3^)	*ρ*_r_ (%)	*GS* (μm)
0.02	5.61	6.02	93	1.3
0.04	5.62	6.04	93	0.8
0.06	5.65	6.06	93	2.0
0.07	5.62	6.08	92	3.5

**Table 2:** Dielectric parameters for BSTM ceramics.

Sample	*x*	*ε*′_m_	*ε*′_RT_	*T*_c_ (°C)	*T*_0_ (°C)	*C* (×10^5^ °C)
BS2TM	0.02	5480	2080	105.5	82.0	1.73
BS4TM	0.04	7620	3030	71.6	57.8	1.77
BS6TM	0.06	14,252	4070	39.5	40.3	1.56
BS7TM	0.07	15,220	13,810	20.4	28.7	1.43
